# The GLP-1-mediated gut-kidney cross talk in humans: mechanistic insight

**DOI:** 10.1152/ajpcell.00476.2023

**Published:** 2023-12-18

**Authors:** Gitte R. Hinrichs, Peter Hovind, Ali Asmar

**Affiliations:** ^1^Department of Nephrology, Odense University Hospital, Odense, Denmark; ^2^Department of Molecular Medicine, Cardiovascular and Renal Research, University of Southern Denmark, Odense, Denmark; ^3^Department of Clinical Physiology & Nuclear Medicine, Bispebjerg-Frederiksberg Hospital, Copenhagen University Hospital, Copenhagen, Denmark; ^4^Department of Clinical Physiology & Nuclear Medicine, Rigshospitalet, Copenhagen University Hospital, Copenhagen, Denmark; ^5^Department of Clinical Medicine, University of Copenhagen, Copenhagen, Denmark

**Keywords:** glomerular filtration rate, GLP-1, kidney, renal blood flow, renin-angiotensin-aldosterone system

## Abstract

Incretin-based therapy is an antidiabetic and antiobesity approach mimicking glucagon-like peptide-1 (GLP-1) with additional end-organ protection. This review solely focuses on randomized, controlled mechanistic human studies, investigating the renal effects of GLP-1. There is no consensus about the localization of GLP-1 receptors (GLP-1Rs) in human kidneys. Rodent and primate data suggest GLP-1R distribution in smooth muscle cells in the preglomerular vasculature. Native GLP-1 and GLP-1R agonists elicit renal effects. Independently of renal plasma flow and glomerular filtration rate, GLP-1 has a natriuretic effect but only during volume expansion. This is associated with high renal extraction of GLP-1, suppression of angiotensin II, and increased medullary as well as cortical perfusion. These observations may potentially indicate that impaired GLP-1 sensing could establish a connection between salt sensitivity and insulin resistance. It is concluded that a functional GLP-1 kidney axis exists in humans, which may play a role in renoprotection.

## Graphical abstract

**Figure:**
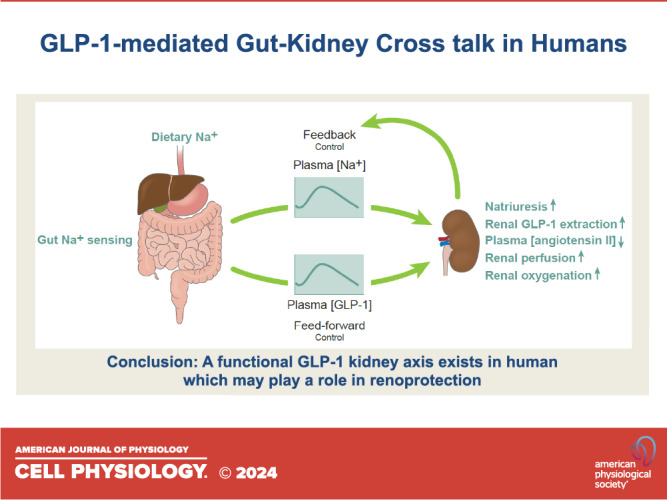

## INTRODUCTION

Glucagon-like peptide-1 (GLP-1) is a 30-amino acid peptide hormone, which is formed from proglucagon and primarily synthesized by enteroendocrine L cells of the intestinal epithelium and in small groups of neurons in the nucleus of the tractus solitaries in the brain stem. GLP-1 is secreted in a nutrient-dependent manner and stimulates insulin secretion, inhibits glucagon release and gastric emptying, and induces satiety, mitigating postprandial glucose increase (1). In contrast to healthy subjects, the so-called incretin effect is substantially reduced or even absent in patients with type 2 diabetes (T2DM) (1), making the GLP-1 receptor (GLP-1R) an attractive target for antidiabetic treatment, using GLP-1 receptor agonists (GLP-1RAs). Moreover, GLP-1RAs cause significant weight loss and represent a new pharmacological antiobesity treatment strategy (2). The GLP-1R is widely expressed in several tissues, including the pancreas, central nervous system, heart, vessels, and kidneys (3–5). In patients with T2DM and a high cardiovascular risk profile, GLP-1RAs exert beneficial cardiovascular and renal effects (6, 7); reduce albuminuria, plausibly beyond the glycemic control; and marginally attenuate estimated glomerular filtration rate (GFR) decline (8, 9). Therefore, the action of GLP1-RAs seems not to be solely restricted to the glucose-lowering effect, and preservation of kidney function in patients with diabetes is presumed; it could be speculated that GLP-1RAs possess distinct protective renal effects.

In rodents, GLP-1R activation increases natriuresis via preglomerular vasodilation and decreases proximal tubular reabsorption of sodium by inhibiting the Na^+^/H^+^ exchanger isoform 3 (NHE-3) (10, 11). Furthermore, an interaction with the myocardium with renal impact has been demonstrated in hypertensive rodents in which GLP-1R activation in the atria leads to atrial natriuretic peptide (ANP) secretion and thereby natriuresis, vasodilation, and systemic blood pressure decrease (12). However, such an effect on ANP has not been demonstrated in humans (13, 14). In humans, the natriuretic effect of GLP-1 is not uncovered at baseline, but when extracellular volume (ECV) is expanded similar to a rather large postprandial electrolyte absorption (13–20), intake of salt appears to directly amplify GLP-1 release versus intake of glucose alone (21). GLP-1-evoked natriuresis is associated with high renal extraction of GLP-1, suppression of angiotensin II (ANGII), and increased renal medullary and cortical perfusion, while it appears to be independent of measurable changes on overall renal plasma flow (RPF) and GFR. The natriuretic action of GLP-1, high renal GLP-1 extraction, and ANGII suppression can be blocked by a GLP-1R antagonist. (13, 15, 22). Differences between species may obviously contribute to explaining these discrepancies; however, in animal experiments (23), GLP-1 levels are typically increased 100-fold above physiological levels compared with a 10-fold increase (slightly supraphysiological levels) investigated in mechanistic human studies. The purpose of this review is to elaborate on possible renal mechanisms of GLP-1 solely in humans, excluding experimental studies in animal models.

## GLP-1 RECEPTOR EXPRESSION AND GLP-1 EXTRACTION IN THE HUMAN KIDNEY

The GLP-1R is a transmembrane receptor, and activation increases cAMP concentration and activations of downstream pathways coupled to protein kinase A (24, 25). The main GLP-1R-expressing compartment is the β–cells in the islets of Langerhans in the pancreas, but GLP-1Rs have also been demonstrated in numerous extrapancreatic tissues. Unfortunately, the identification of GLP-1Rs is challenged by the lack of antibodies with sufficient sensitivity and specificity, explaining the limited data for human kidneys. By using the pancreatic form of human GLP-1R cDNA as a probe, Wei et al. (26) in 1995 detected mRNA from human kidneys, and in 2007, Körner et al. (3) investigated nonneoplastic human tissues resected along with tumor tissue from multiple samples (*n* = 209) and analyzed the GLP-1R expression in several organs by receptor autoradiography. In the kidney, they reported that GLP-1Rs were present in small amounts in hilar and intralobular arteries (*n* = 2). Schlatter et al. (5) analyzed one human sample from a patient undergoing nephrectomy for renal cancer. By immunohistochemistry (IHC) of this human kidney cortex, they revealed overlapping staining of dipeptidyl peptidase-4 (DPP-4) and GLP-1R, indicating predominantly expression in proximal tubular cells. Moreover, they confirmed GLP-1R expression at the protein level by Western blot, with a band at approximately 63 kDa. Subsequently, doubts have arisen regarding the sensitivity of commercial antibodies against GLP-1Rs (27). In 2014, Pyke et al. (4) developed a primate-specific and highly sensitive monoclonal GLP-1R antibody for IHC and observed the expression of GLP-1Rs only in smooth muscle cells (SMCs) in the arterial and arteriolar walls in the cortex of 2 postmortem human samples of normal kidney, in accordance with expression in the preglomerular vasculature (4).

In summary, data are sparse on the localization of the GLP-1R expression in the human kidney, and it has primarily been demonstrated in the SMC of the preglomerular vasculature. Moreover, to our knowledge, no single-cell RNA has yet been found in the human kidney (28); however, data are still scarce.

In healthy humans and patients with T2DM without nephropathy, GLP-1 is extracted by ∼45% in the kidney, which exceeds what can be explained by glomerular filtration (13, 29, 30). Since GLP-1Rs have been localized to the preglomerular vasculature, binding to these receptors with subsequent internalization could explain the additional extraction. This is supported by a previous human study in which the GLP-1R was successfully antagonized, demonstrating a significant reduction in extraction to ∼25%, which is sufficient to account for what can be explained by glomerular filtration. This is in line with native GLP-1 being freely filtered in the glomerulus (15). The GLP-1-degrading enzyme DPP-4 activity in the renal vasculature cannot explain the arterial and renal venous concentration difference, since this was also seen for total GLP-1 concentrations, which is not affected by DPP-4 degradation (1). However, DPP-4 degradation may contribute additionally since the extraction ratio was slightly higher for intact compared with total GLP-1 (13, 29, 30).

## RENAL HEMODYNAMIC AND NATRIURETIC EFFECTS OF GLP-1 UNDER PHYSIOLOGICAL AND PHARMACOLOGICAL CONDITIONS

Despite a significant renal extraction of GLP-1 and distribution of GLP-1Rs in the afferent arteriole, administration of native GLP-1 in humans does not affect RPF nor GFR under physiological conditions (13, 16, 18). These results contradict other studies in which GLP-1RAs under elevated concentrations increase RPF and GFR in overweight but otherwise healthy participants, pointing to preglomerular vasodilation, partially nitric oxide dependent, together with an independent natriuretic effect (17). This preglomerular vasodilatory effect was absent in patients with T2DM, perhaps associated with impaired vascular function and/or downregulation of the GLP-1R, whereas the natriuretic effect remained (20). Altogether, these findings indicate that the renal hemodynamic effects of GLP-1 are dose dependent and that the hemodynamic and natriuretic effects involve different mechanisms. In line with this, human studies demonstrate the natriuretic effect of native GLP-1 in volume-expanded participants under physiological and pharmacological situations, independent of RPF and GFR (13–20). Interestingly, when the ECV was not expanded, it was not possible to demonstrate a GLP-1-induced natriuretic response (29, 30). Data so far indicate that physiologically relevant changes in circulating GLP-1 elicit a fast-acting increase in renal sodium excretion during saline loading, probably elicited via volume-regulating mechanisms.

In 2004, Gutzwiller et al. (16) demonstrated that 3-h GLP-1 infusion dose dependently increased renal sodium excretion by around 60% in healthy participants as well as in obese, insulin-resistant males with mild glomerular hyperfiltration at baseline. Interestingly a simultaneous decrease in GFR (determined by endogenous creatinine) by around 6% (from 151 ± 8 to 142 ± 8 mL/min) was only seen in participants with glomerular hyperfiltration at baseline. Due to a simultaneous decrease in renal hydrogen excretion by ∼75%, it was suggested that GLP-1 decreases proximal tubular reabsorption of sodium by inhibiting NHE-3, leading to a decrease in GFR possibly via the tubuloglomerular feedback mechanism. This was subsequently supported by Skov et al. (18) in healthy male participants, demonstrating that GLP-1 increased renal sodium clearance concomitantly with an increase in renal lithium clearance. Under fixed sodium intake, it is generally accepted that the renal clearance and extraction of lithium correspond to the fractional sodium reabsorption in the proximal tubule. The natriuretic effect was associated with a decrease in plasma ANGII levels but not with changes in renin, aldosterone, or urinary excretion of angiotensinogen. In accordance with Gutzwiller et al. (16), Skov et al. (18) could not demonstrate any effect of GLP-1 on renal hemodynamics in healthy subjects without glomerular hyperfiltration measured by constant infusions of ^51^Cr-EDTA and ^123^I- hippuran coupled with urinary sampling to measure GFR and RPF, respectively. In both studies, a natriuretic response during increased plasma levels of GLP-1 was observed under excessive load of hypertonic (2.5% NaCl, 0.03 mL·min^−1^·kg^−1^ for 180 min) or isotonic saline (0.9% NaCl, 750 mL·h^−1^ for 7 h), suggesting that GLP-1 may contribute to natriuresis via volume-mediated mechanisms independent of renal hemodynamics. This hypothesis was further confirmed by Asmar et al. (13, 29), demonstrating that expanded ECV uncovered a twofold increase in natriuresis independent of total RPF and GFR, pointing to a functional GLP-1 kidney axis in man. This natriuretic action was, however, not associated with increased flow out of the proximal tubule during GLP-1 infusion as assessed by lithium clearance nor by increased urine flow, hydrogen excretion, or free water clearance. Although indirect, these data point to a natriuretic effect of GLP-1 mediated by more distal nephron segments. In addition, subsequent studies suggest that GLP-1-induced natriuresis is dependent on GLP-1R activation and associated with ANGII suppression, possibly leading to increases in predominantly medullary but also cortical perfusion in healthy human kidney (13, 15, 22).

## EFFECTS OF GLP-1 ON CENTRAL HEMODYNAMICS AND AUTONOMIC NERVOUS ACTIVITY

It is unlikely that pressure-mediated mechanisms are involved in the natriuretic effect of GLP-1. Acute infusions demonstrate inconsistent data in humans, having no effects on blood pressure (18), and even when acute GLP-1 administration increases systolic blood pressure, this is not associated with natriuresis (29). However, cardiac output is simultaneously increased, proportionally greater than the increase in mean arterial pressure due to vasodilation in vascular beds (skeletal muscle and adipose tissue) except for net renal hemodynamics (29, 31). Together with an increase in cardiac work and thereby blood flow, this can explain the increase in cardiac output in healthy lean subjects.

Chronic administration of GLP-1RAs, however, reduces systolic blood pressure slightly (≈2 mmHg) at least when evaluated as a secondary endpoint in glycemia-lowering studies while simultaneously increasing heart rate (32).

Since GLP-1 powerfully inhibits central nervous parasympathetic outflow in humans, another possibility is that the effects of GLP-1 involve inhibition of vagal activity, which would also result in an increased heart rate (33). However, it was not possible to demonstrate changes in heart rate variability during acute GLP-1 infusions (29, 30). Also, the degree of physiological hyperinsulinemia during GLP-1 infusions most likely did not affect sympathetic nervous activity as seen during high physiological/pharmacological concentrations of insulin accompanied by sympathoexcitatory effects determined by elevations in plasma noradrenaline concentrations and increased muscle sympathetic nerve activity (34, 35). During GLP-1 infusions, plasma catecholamine concentrations were unaffected (29, 30). Alternatively, the increase in heart rate may be due to activation of GLP-1Rs located perhaps in the sinoatrial node (4).

## GLP-1 AND THE RENIN-ANGIOTENSIN-ALDOSTERONE SYSTEM

In a study in which ECV was expanded with isotonic saline infusion to elucidate the natriuretic effect of GLP-1, plasma renin concentrations decreased significantly (13). This effect was presumably mediated by the volume load. Nevertheless, since renin acts as the major rate-limiting step in the renin-angiotensin-aldosterone system (RAAS), it was noteworthy that ANGII only decreased significantly during the GLP-1-induced natriuretic action, independently of the remaining RAAS components as well as ANP and brain natriuretic peptide. The mechanisms for suppression of ANGII are still not clarified, but recently it has been demonstrated that GLP-1-dependent ANGII suppression, high renal GLP-1 extraction, and GLP-1’s natriuretic effect all depend specifically and fully on GLP-1R activation independent of changes in total RPF and GFR (13, 15). Our preliminary data suggest constant levels of circulating angiotensinogen, which therefore cannot explain the GLP-1-mediated ANGII suppression. Data indicate that GLP-1 enhances pathways of ANGII degradation rather than inhibiting its formation. The exclusive suppression of ANGII may take place via direct inhibition of ACE-mediated ANGII synthesis in the kidney tissue, where all components of the RAAS are present. Clinical studies of GLP-1 with ANGII clamps are needed to clarify whether GLP-1 is involved in the regulation of ANGII half-life and whether this involves other factors [e.g., availability of activity of angiotensin-converting enzyme (ACE) and increased activity of ACE-2 and neprilysin with enhanced clearance and metabolism of ANGII, including the ACE2/angiotensin-(1–7)/MAS axis] that influence ANGII levels.

**Figure 1.**
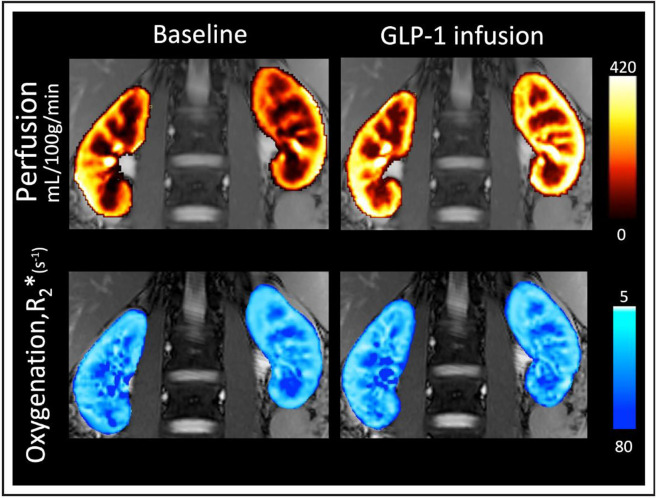
Perfusion and oxygenation (R_2_*) maps for 1 participant immediately before (baseline) and 15 minutes after commencement of glucagon-like peptide 1 (GLP-1) infusion (22). Significant increases in both medullary and cortical perfusion were observed, as was either stable or slightly improved oxygenation (similar or reduced R_2_* values).

From a pathophysiological perspective, it is interesting that circulating and intrarenal ANGII generally promote the progression of diabetic nephropathy and hypertensive kidney diseases through proinflammatory and growth-promoting effects (36, 37). The direct GLP-1-mediated suppression of ANGII in humans possibly accounts for an increase in mainly renal medullary perfusion but also renal cortical perfusion and renal oxygenation in the healthy human kidney during saline loading (22) (Fig. 1). Renal hypoxia plays a central role in the pathway for most etiologies of chronic kidney disease, e.g., microvascular damage and excessive renal energy expenditure due to hyperglycemia-related glomerular hyperfiltration and associated increased sodium reabsorption pathway (38). Thus, by preserving renal tissue oxygenation, improved renal perfusion may contribute to the long-term beneficial cardiovascular and renal outcomes of GLP-1RAs.

## GUT SODIUM SENSING: A GLP-1-MEDIATED FEED-FORWARD NATRIURETIC EFFECT

**Figure 2.**
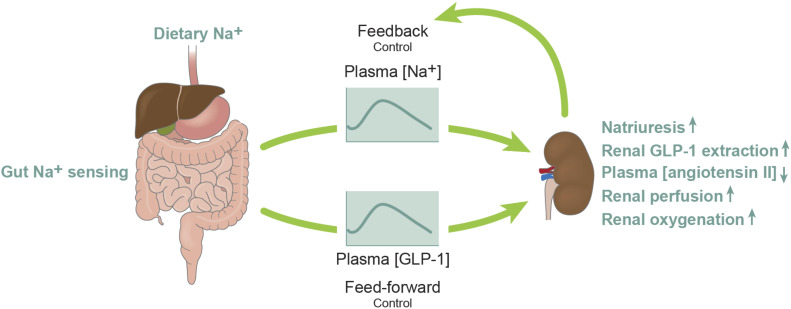
The glucagon-like peptide-1 (GLP-1)-mediated gut-kidney cross talk in humans. Mechanistic studies conducted in healthy humans demonstrate a GLP-1-mediated gut-kidney cross talk in which sodium intake is sensed in the gut, leading to higher postprandial GLP-1 secretion. The expansion of extracellular fluid volume uncovers a natriuretic action of GLP-1. The feed-forward natriuretic system is associated with high renal extraction of GLP-1, suppression in circulating ANGII levels, and increased renal medullary and cortical perfusion and oxygenation.

While the kidneys regulate sodium balance and blood pressure, the gastrointestinal (GI) tract has taste- and nutrient-sensing receptors and sensors for electrolytes partly coupled to the release of GI hormones (39). Previous studies support the involvement of a gut-kidney axis in the excretion of a dietary sodium load mediated by GI hormones, including gastrin and cholecystokinin (CCK), possibly through interaction with renal dopamine receptors (40–42). Thus there is increasing evidence of the importance of the GI tract in blood pressure regulation potentially through feed-forward effects on renal sodium handling (Fig. 2).

As GLP-1 release is physiologically regulated by luminal stimuli in the GI-tract, and since dietary salt intake varies strongly within and between individuals, a direct salt sensitivity of L-cell GLP-1 release could provide a sequential, feed-forward signal that adds to the gastric/duodenal signals from gastrin/CCK for renal sodium excretion. GLP-1 secretion from the intestines normally increases following nutrient intake, and dietary carbohydrates are major determinants of GLP-1 secretion (43). There is evidence that the main mechanism behind this is glucose uptake in the enteroendocrine L cells mediated via sodium-glucose cotransporter 1 (SGLT1) (43, 44). Under physiological circumstances, it would not be expected that sodium availability is rate limiting for glucose absorption and thereby GLP-1 secretion due to the high abundance of sodium in the diet and the bile and gastropancreatic secretions. The addition of dietary salt to a meal increases the osmolarity of the meal, which could be a contributory factor to the increased GLP-1 secretion mediated by the electrogenic nature of Na^+^-coupled glucose uptake by SGLT1, highly expressed in L cells (43). Thus SGLT1 is an L-cell glucose sensor, utilizing the inwardly directed sodium gradient to drive glucose influx, which in turn stimulates GLP-1 secretion to the circulation. Data from human studies indicate that SGLT1 may indirectly act as an L-cell sodium sensor in which increased dietary, and thus luminal sodium amplifies glucose/sodium-dependent GLP-1 secretion (21) (Fig. 2).

In perspective, GLP-1 could potentially support a positive feed-forward natriuresis that may partly be associated with lower ANGII levels and higher renal perfusion. This mechanism could have significant implications for renal sodium handling in health and disease since there is an impaired incretin secretion and action in T2DM and obesity. Thus impaired GLP-1 sensing may link salt sensitivity with insulin resistance, increased sympathetic nervous activity, and ultimately the development of hypertension (45).

Besides potent glucose-lowering actions, GLP-1RAs improve body weight, blood pressure, and dyslipidemia, and cardiovascular outcome trials demonstrate beneficial cardiovascular actions of GLP-1RAs used in patients with T2DM at high cardiovascular risk (2, 6, 7). The GLP-1RAs are safe to use in patients with diabetic kidney disease, and in addition to standard treatment of diabetes, they modestly reduce albuminuria, plausibly beyond the effects of glycemic control, and halt estimated GFR decline in patients with a high risk of cardiovascular and renal events (46). It remains uncertain whether GLP-1RAs directly improve hard renal outcomes. However, this is currently being addressed by the FLOW trial by Novo Nordisk, which compares GLP-1RA versus placebo in patients with T2DM and chronic kidney disease and has recently been stopped early due to efficacy. Thus the beneficial cardiovascular effects of GLP-1 may partly be related to renoprotection and might represent the restoration of the gut-kidney cross talk (Fig. 2).

## LIMITATIONS

In the present mechanistic review, most human studies were conducted under acute elevation of either slightly supraphysiological or “pharmacological” plasma GLP-1 concentrations for a few hours. Obviously, this does not necessarily reflect heavily engineered long-acting GLP-1RAs used in a larger population with T2DM, and improvements in kidney function can plausibly also involve changes in glycemia, dyslipidemia, and cardiovascular function. Conversely, acute onset mechanistic changes are likely not detectable in a long-term clinical trial setup.

## DISCLOSURES

Dr. A. Asmar consulted for Novo Nordisk. No conflicts of interest, financial or otherwise, are declared by the remaining authors.

## AUTHOR CONTRIBUTIONS

A.A. conceived and designed research; A.A. prepared figures; G.R.H., P.H., and A.A. drafted manuscript; G.R.H., P.H., and A.A. edited and revised manuscript; G.R.H., P.H., and A.A. approved final version of manuscript.
